# Identification and characterization of NAGNAG alternative splicing in the moss *Physcomitrella patens*

**DOI:** 10.1186/1471-2229-10-76

**Published:** 2010-04-28

**Authors:** Rileen Sinha, Andreas D Zimmer, Kathrin Bolte, Daniel Lang, Ralf Reski, Matthias Platzer, Stefan A Rensing, Rolf Backofen

**Affiliations:** 1 Bioinformatics group, University of Freiburg, Georges-Koehler-Allee 106, 79110 Freiburg, Germany; 2 Faculty of Biology, University of Freiburg, Hauptstrasse 1, 79104 Freiburg, Germany; 3 Plant Biotechnology, Faculty of Biology, University of Freiburg, Schaenzlestrasse 1, 79104 Freiburg, Germany; 4 Freiburg Initiative for Systems Biology (FRISYS), University of Freiburg, Schaenzlestrasse 1, 79104 Freiburg, Germany; 5 Centre for Biological Signalling Studies (bioss), University of Freiburg, Albertstr. 19, 79104 Freiburg, Germany; 6 Genome Analysis, Leibniz Institute for Age Research - Fritz Lipmann Institute, Beutenbergstr. 11, 07745 Jena, Germany; 7 Philipps-Universität Marburg, Laboratorium für Zellbiologie, Karl-von-Frisch Str., 35032 Marburg, Germany

## Abstract

**Background:**

Alternative splicing (AS) involving tandem acceptors that are separated by three nucleotides (NAGNAG) is an evolutionarily widespread class of AS, which is well studied in *Homo sapiens *(human) and *Mus musculus *(mouse). It has also been shown to be common in the model seed plants *Arabidopsis thaliana *and *Oryza sativa *(rice). In one of the first studies involving sequence-based prediction of AS in plants, we performed a genome-wide identification and characterization of NAGNAG AS in the model plant *Physcomitrella patens*, a moss.

**Results:**

Using Sanger data, we found 295 alternatively used NAGNAG acceptors in *P. patens*. Using 31 features and training and test datasets of constitutive and alternative NAGNAGs, we trained a classifier to predict the splicing outcome at NAGNAG tandem splice sites (alternative splicing, constitutive at the first acceptor, or constitutive at the second acceptor). Our classifier achieved a balanced specificity and sensitivity of ≥ 89%. Subsequently, a classifier trained exclusively on data well supported by transcript evidence was used to make genome-wide predictions of NAGNAG splicing outcomes. By generation of more transcript evidence from a next-generation sequencing platform (Roche 454), we found additional evidence for NAGNAG AS, with altogether 664 alternative NAGNAGs being detected in *P. patens *using all currently available transcript evidence. The 454 data also enabled us to validate the predictions of the classifier, with 64% (80/125) of the well-supported cases of AS being predicted correctly.

**Conclusion:**

NAGNAG AS is just as common in the moss *P. patens *as it is in the seed plants *A. thaliana *and *O. sativa *(but not conserved on the level of orthologous introns), and can be predicted with high accuracy. The most informative features are the nucleotides in the NAGNAG and in its immediate vicinity, along with the splice sites scores, as found earlier for NAGNAG AS in animals. Our results suggest that the mechanism behind NAGNAG AS in plants is similar to that in animals and is largely dependent on the splice site and its immediate neighborhood.

## Background

Eukaryotic primary mRNAs consist of protein-coding regions (exons) and intervening non-coding regions (introns). The mature mRNA transcript, which acts as substrate for translation into protein, is produced by removing introns in a process called splicing. Splicing can be either constitutive, always producing the same mRNA, or alternative, via variable inclusion of parts of the primary transcript. Alternative splicing (AS) is thus a mechanism that enables multiple transcripts and proteins to be encoded by the same gene, thereby promoting transcript and protein diversity [1]. Furthermore, events of AS can provide an additional level of post-transcriptional gene regulation, e.g. by the production of mRNA isoforms with truncated open reading frames that are subject to degradation by the nonsense mediated decay pathway [2,3]. AS is particularly widespread in higher eukaryotes, especially in mammals - it has been estimated that up to 94% of all multi-exonic *H. sapiens *genes are alternatively spliced [4]. Large-scale detection of AS usually involves expressed sequence tags (ESTs), microarray, or RNA-seq analysis. However, not all AS events can be detected by these methods. Moreover, nowadays genomic sequence data is being churned out at a much faster rate than transcript data, that is, many genomes have low transcript coverage. Thus, there is a need for independent methods of detecting AS.

**Figure 1:**
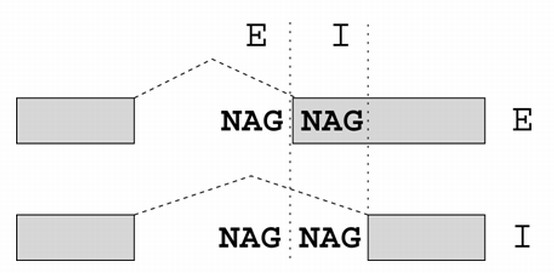
**NAGNAG alternative splicing**. Nomenclature of NAGNAG AS with E and I sites and isoforms.

It has been shown that AS involving alternative donors/acceptors separated by 2-12 nt, also called "subtle alternative splicing" (due to the small difference in length in the transcript isoforms), is an evolutionarily widespread class of AS among animals, and among these, NAGNAG AS, involving acceptors separated by 3 nt, is the most common [5-8]. The terminology "NAGNAG" refers to events of AS that involve two acceptors (two "AG"s) which are preceded by any of the four possible nucleotides (N = A, C, G or T). Hence, the generic pattern for such tandem splice sites is "NAGNAG". NAGNAG AS can result in one of three possibilities (Figure 1) - constitutive use of the first acceptor (the so-called exonic, or "E" variant), constitutive use of the second acceptor (the so-called intronic, or "I" variant), or use of both acceptors, that is, alternative splicing (the "EI" variant) [5]. NAGNAGs contribute 45% of all conserved alternative acceptors in *H. sapiens *and *M. musculus *[9]. Since the difference between the two isoforms is three nucleotides, no frameshift is induced, and the usual impact of a NAGNAG AS event is the insertion or deletion of one amino acid. In a recent study, we predicted the splicing outcome at NAGNAG acceptors in seven animal genomes (human, mouse, rat, dog, chicken, fruit fly and nematode) with a high degree of accuracy, and 83% of the experimentally validated cases agreed with the predictions [10]. In agreement with previous studies [11,12] this indicated that the mechanism behind NAGNAG AS seems to be simple, stochastic and conserved in vertebrates and beyond.

While there have been numerous experimental as well as computational studies of AS in animals, the study of AS in plants is still in its early stages [13]. Although AS is commonly observed in plants, the overall abundance of AS seems to be lower than in animals. Several studies have estimated that between 20%-30% of plant genes undergo AS [14-17], while the current estimate based on deep sequencing of the *Arabidopsis thaliana *transcriptome is 42%-56% of intron-containing genes [18]. EST-based detection of AS in plants revealed that intron retention appears to be the most common kind of AS event in plants [13-16]. Exon-skipping, which is the most common event in animals [19], is much less frequent in plants [14,16]. The two prevalent models for spliceosome assembly are intron-definition, which applies to short introns (thus to a majority of plant introns) and involves the intron as the initial unit of recognition during spliceosome assembly; and exon-definition, which applies to long introns introns (thus to a majority of animal introns), and involves recognition of the exon as the initial unit for splicing [14,20-22]. Thus, one would expect inaccurate splicing to result in intron-retention under the intron-definition model, and exon-skipping under the exon-definition model [13]. Hence the results showing that intron-retention is the most common AS event in plants and exon-skipping in animals are consistent with these models of splicing. However, alternative acceptors and donors seem to occur at a comparable frequency [16]. In particular, short distance or subtle AS events, seem to be just as common, and NAGNAG acceptors are widespread and abundant; a study on AS found 953 alternative NAGNAGs in rice and 485 in *A. thaliana *[16].

Initial analyses of the model plant *P. patens*, the first sequenced bryophyte, indicated a distribution of AS events similar to other plants studied so far [23]. Consequently, we here aimed to characterize and predict the extent of NAGNAG AS in *P. patens*. Analysis of the available transcript data indicates that NAGNAG AS is just as common in the moss *P. patens *as in seed plants. We achieved a high level of performance *in silico*, and 64% of the cases of well-supported AS using independently generated 454 data could be correctly predicted. In agreement with a recent study comparing *A. thaliana *and *O. sativa *with mammals [24], our results suggest that the mechanism of NAGNAG AS is similar in plants and animals.

## Results and discussion

### Identification of alternative NAGNAGs using Sanger ESTs

**Figure 2:**
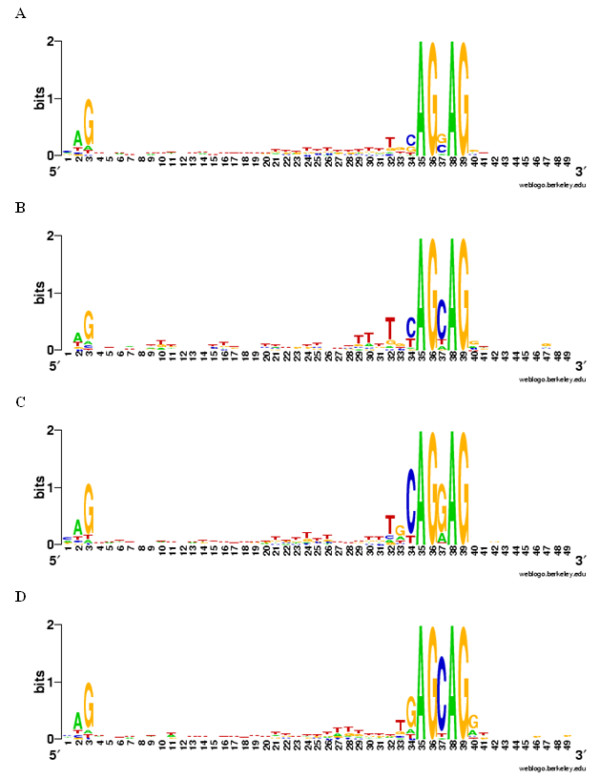
**Sequence logos of NAGNAG splice sites**. The first three positions represent the last 3 nucleotides (nt) of the upstream exon, followed by the 30 nt upstream of the NAGNAG, the NAGNAG motif itself, and the 10 nt downstream of the NAGNAG (total 49 positions). A: all splice sites; B: EI sites, C: E sites; D: I sites.

Since the extent of NAGNAG occurence in *P. patens *had not been reported, we sought to identify genomic NAGNAGs. To find all genomic NAGNAGs (constitutive as well alternative), we looked for all annotated intron-exon boundaries which had an AG at three positions upstream or downstream of the annotated acceptor. This yielded 9,427 NAGNAG motifs, of which 5,031 were covered by Sanger ESTs (Additional file 1). Cases where the EST evidence supported only one of the NAG acceptors were called constitutive, whereas cases with EST support for both acceptors were called alternative (here EST support means at least one EST from a high quality alignment, as described in the section "Methods"). 295 (5.9%) of the detected 5,031 NAGNAGs with Sanger EST coverage were alternatively spliced (EI form), while 2,695 (53.6%) were exclusively spliced at the first (intron proximal) acceptor (E form, i.e. part of the NAGNAG is exonic) and 2,041 (40.5%) were spliced only at the second (intron distal) acceptor (I form, i.e. the entire NAGNAG is intronic). Thus, NAGNAG AS is common in *P. patens*. Sequence logos for all NAGNAG splice sites as well as for EI, E and I sites are visualized in Figure 2.

### Gene ontology enrichment analysis

To assess whether genes with NAGNAG AS in *P. patens *are enriched for specific functional categories and whether there is any similarity with *A. thaliana *and *O. sativa *in that sense, we analyzed Gene Ontology (GO) term annotations with GOSSIP [25]. GO terms with a FDR corrected p-value (q-value) less than 0.05 were considered significantly different. We found that 42 genes with the term plastid (GO:0009536, q-value 0.043) are statistically enriched (Table 1) in the set of *P. patens *genes with EST support for an alternative NAGNAG acceptor (225 genes). This could be confirmed by the GOSSIP analysis for the *P. patens *alternative NAGNAG genes supported by Sanger and 454 reads (498 genes, q-value: 8.35E-04). In addition, the terms organelle and mitochondrion (Table 1) were found to be enriched among the NAGNAG genes in *P. patens*. "DNA binding" (GO:0003677) which is reported for *A. thaliana *and *O. sativa *to be enriched in alternative NAGNAG genes [24], could not be observed for *P. patens*. To further examine this inconsistency, the supported alternative NAGNAG genes from *A. thaliana *(combined gene set from [26] and [24]) were subjected to GO enrichment analysis as well (Table 1). This analysis confirms the term "DNA binding" as overrepresented among the *A. thaliana *NAGNAG genes (q-value: 2.28E-04), thus consistent with the analyses for *A. thaliana *and *O. sativa *[24] as well as for mouse [12]. However, "DNA binding" was not found to be enriched in *P. patens *NAGNAG genes. In contrast to the analyses for the seed plants and mouse, Fisher's exact test with false discovery rate corrected p-values was used here, instead of a chi-square test. However, if the parent term of DNA binding, nucleotide binding (GO:0000166) was also subjected to a chi-square test for *P. patens*, it was found to be enriched (p = 0.02). The fact that "DNA binding" was not found to be enriched in *P. patens *NAGNAGs might be due to the current status of the *P. patens *annotation (v1.2) - e.g. in many cases the gene models lack 5' and 3' regions and therefore do not cover the whole protein sequence. On the other hand, mosses and vascular plants diverged more than 450 million years ago and thus *P. patens *alternative NAGNAG acceptor genes might be different. Nevertheless, the GO enrichment analysis in terms of the category "cellular component" reveals that *A. thaliana *as well as *P. patens N*AGNAG genes share a bias towards the term "intracellular organelle", which includes "nucleus" and "plastid" (Table 1). In addition to the enriched molecular function "DNA binding", our analysis confirmed the functions "RNA binding", "transcription factor activity" and "transcription regulator activity" to be also slightly enriched in *A. thaliana *alternative NAGNAG acceptor genes (Table 1), which is coherent with reports for *M. musculus *[12].

**Table 1:** GO analyses of genes with alternative NAGNAG acceptor site

Organism/subset	Name	GO Term	FDR (q-value)	# in test group	# in reference group	# non annot. test	# non annot. reference group
***P. patens *alternative NAGNAG genes Sanger support**	cytoplasmic part	GO:0044444	0.0133	78	5407	100	11894
	plastid	GO:0009536	0.04291	42	2607	136	14694
	membrane-bound organelle	GO:0043227	0.04291	89	6778	89	10523
	intracellular membrane-bound organelle	GO:0043231	0.04291	89	6778	89	10523
	intracellular part	GO:0044424	0.04963	105	8388	73	8913
***P. patens *alternative NAGNAG genes Sanger and/or 454 support**	cytoplasmic part	GO:0044444	3.52E-04	168	5317	232	11762
	plastid	GO:0009536	8.35E-04	90	2559	310	14520
	membrane-bound organelle	GO:0043227	8.35E-04	196	6671	204	10408
	intracellular membrane-bound organelle	GO:0043231	8.35E-04	196	6671	204	10408
	intracellular part	GO:0044424	0.00379	229	8264	171	8815
	intracellular	GO:0005622	8.35E-04	251	9029	149	8050
	cytoplasm	GO:0005737	3.78E-04	191	6312	209	10767
	mitochondrion	GO:0005739	0.04193	53	1553	347	15526
	thylakoid	GO:0009579	0.02355	12	187	388	16892
	organelle	GO:0043226	0.00436	207	7373	193	9706
	intracellular organelle	GO:0043229	0.00436	207	7373	193	9706
***A. thaliana *alternative NAGNAG genes combined (Iida et al., 2008 and Schindler et al., 2008)**	plastid	GO:0009536	0.0474	50	3021	192	17594
	membrane-bound organelle	GO:0043227	2.28E-04	113	6959	129	13656
	intracellular membrane-bound organelle	GO:0043231	2.28E-04	113	6959	129	13656
	intracellular	GO:0005622	5.17E-05	136	8421	106	12194
	organelle	GO:0043226	1.73E-04	119	7287	123	13328
	intracellular organelle	GO:0043229	1.73E-04	119	7287	123	13328
	nucleic acid binding	GO:0003676	5.98E-06	71	3230	171	17385
	nucleus	GO:0005634	2.02E-04	51	2342	191	18273
	DNA binding	GO:0003677	2.28E-04	49	2250	193	18365
	intracellular part	GO:0044424	2.82E-04	124	7881	118	12734
	cell part	GO:0044464	0.0027	159	11257	83	9358
	RNA binding	GO:0003723	0.01	15	506	227	20109
	binding	GO:0005488	0.01174	132	9231	110	11384
	nucleobase, nucleoside, nucleotide and nucleic acid metabolic process	GO:0006139	0.01314	47	2608	195	18007
	transcription factor activity	GO:0003700	0.01314	33	1646	209	18969
	transcription regulator activity	GO:0030528	0.04363	34	1852	208	18763

### Evolutionary conservation of NAGNAG splicing among plants?

Seven clusters of homologous genes with AS at NAGNAG acceptors in the same intron were reported to be conserved between *A. thaliana *and *O. sativa *[24]. In order to check whether AS NAGNAG events are conserved between *A. thaliana *and *P. patens*, a BLAST based single linkage clustering was performed, using all transcripts with a Sanger-supported NAGNAG. Altogether, 1,088 clusters containing *A. thaliana *and *P. patens *genes were identified, of which five clusters contained genes with a NAGNAG motif at the orthologous (as evidenced by numbering from the transcription start site) intron. Five out of the seven *P. patens *genes in these clusters were selected for experimental validation. In all cases only one of the two isoforms could be detected, which is consistent with the support by Sanger ESTs, which in all cases supported only one of the two isoforms. In addition, in all cases the NAGNAG motif itself is not conserved between *A. thaliana *and *P. patens *(Table 2). In *A. thaliana*, only two of the NAGNAG motifs contains a GAG, whereas four of five in *P. patens *contain a GAG, and are therefore unlikely to represent alternative NAGNAGs ([5,10,12,27], and section below). Given the assumption that we are looking at orthologous or at least homologous positions and our transcript evidence is sufficient, this observation can be explained by two possible evolutionary scenarios. In the first scenario the alternative NAGNAG sites are ancestral and have diverged in the lineages leading to *A. thaliana *and *P. patens*. While they might have been inactivated by the introduction of a GAG in the moss *P. patens*, they have been retained functional in *A. thaliana*. In the second scenario these alternative NAGNAG acceptors in *A. thaliana *arose after the divergence of mosses and seed plants. Given the current scarce data, both scenarios appear equally parsimonious. In order to decide which scenario is true, additional taxa would have to be included into the analysis. Given the current data and analyzes there is evidence for conserved NAGNAG AS events between *O. sativa *and *A. thaliana*, but not between *P. patens *and *A. thaliana*. Thus, it appears as if NAGNAG AS is not conserved across several hundreds of millions of years [28] or arose secondarily.

**Table 2:** NAGNAG motifs occuring atconserved positions in *A. thaliana *and *P. patens*

*A. thaliana*		*P. patens*		
At5g65010	TCTTG**TAGGAG**GGC	Phypa_180723	GGCAA**CAGGAG**GGC	**validated**
At5g06600	TTTTG**CAGCAG**CCA	Phypa_180457	TGTGG**CAGGAG**GAC	
At5g06600	TTTTG**CAGCAG**CCA	Phypa_216093	TGTGG**CAGGAG**GAT	
At5g12210	TTTGC**TAGAAG**AAA	Phypa_191544	ACATT**CAGGAG**GAT	
At2g35520	TGATT**GAGCAG**GTT	Phypa_74146	CTGGG**AAGCAG**GTG	

At3g06550	TATGT**TAGTAG**GCA	Phypa_226366	TAGAG**AAGCAG**GTG	**not validated**
At3g06550	TATGT**TAGTAG**GCA	Phypa_65220	GGAAT**GAGCAG**GTG	

### Prediction of NAGNAG AS in *P. patens*

The most crucial prerequisite for good prediction performance is a reliable training dataset. It is critical that the samples are correctly labelled as far as possible. In terms of datasets of alternative and constitutive exons, this means that we should use the available transcript evidence judiciously, in order to minimise mislabelling. In other words, we want to avoid the contamination of the set of constitutive exons by alternative exons which currently lack transcript support for being alternative, as well of alternative exons by potentially erroneously labelled exons. Thus, we used filters on the transcript support to improve the reliability of the labels - as in our previous work on NAGNAG AS prediction in animals [10], a training set was constructed based on the following criteria:

(i) constitutive: ≥ 10 ESTs supporting either E or I variant, 0 for the other;

(ii) alternative: ≥ 2 ESTs supporting each variant, ≥ 10% of ESTs supporting minor variant.

**Figure 3:**
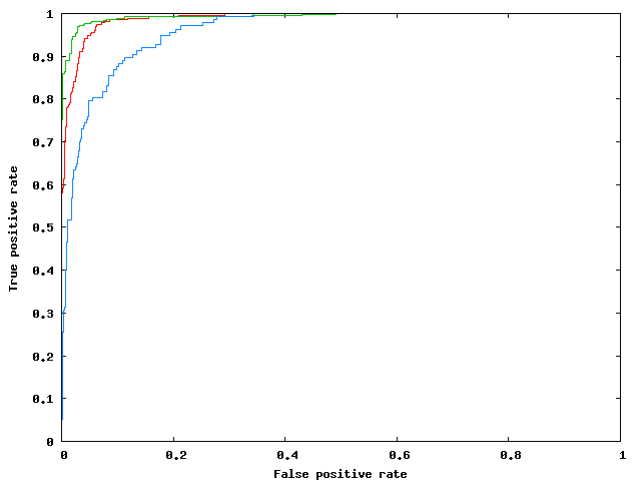
**ROC plot**. Depicting the *in silico *performance on the 3-class [I-class (red), E-class (green), and EI-class (blue)] classification problem. The EI-class, or AS, harder to predict (AUC = 0.96) than the two constitutive variants, E and I (AUC = 0.99 for both).

This yielded a training dataset of 833 NAGNAGs - 696 constitutive (424 E, 272 I) and 137 EI, or alternative cases. The classifiers were trained using this dataset. The remaining 4,198 NAGNAGs (2,271 E, 1,769 I, 158 EI) were used as a test set. It is noteworthy that the average coverage per constitutive NAGNAG in this set is only three ESTs (for both E as well as I cases), indicating that there are potentially many undiscovered alternative NAGNAGs in *P. patens*. The training data was used with a classifier (we used naïve Bayes classifiers, Bayesian networks, and support vector machines, all of which yielded very similar performance) in a cross-validation setting. Briefly, the classifier uses part of the training data to learn a model based on the sample labels and the features, and then uses this model to assign posterior probabilities (P(EI), P(E) and P(I) according to the three possible classed) to each sample. The predicted NAGNAG class is the one which receives the maximum score or posterior probability from the classifier. We computed the receiver operating characteristics (ROC), which is a plot of the true positive rate versus the false positive rate, and measured the area under the ROC curve (AUC), which is a standard measure of the quality of a classifier [29]. An ideal classifier, which makes no errors, would achieve an AUC of 1.0. We used 31 features, and achieved an *in silico *performance of AUC = 0.96, 0.99 and 0.98 for the EI, E and I forms, respectively (Figure 3). This performance was obtained under various cross-validation settings (2-fold, 5-fold, 10-fold, leave-one-out - where n-fold cross-validation means that (n-1)/n of the dataset is used to learn, and the remaining 1/n for prediction - this is repeated n times, and the average performance is reported).

### Generation of additional transcript evidence

As mentioned above, average transcript support was found to be low. In order to generate more evidence for alternative acceptors, next generation sequencing was carried out. For this purpose, adult gametophores carrying gametangia (for review of moss tissues see: [30]) were grown, as this tissue was not well represented in the pre-existing ~400,000 Sanger reads. In addition, the cDNA was normalized in order to equalize transcript abundance and thus avoid redundancy. While the ~400,000 Sanger reads map to 19,186 gene models, the ~600,000 454 reads map to 20,161 gene models. The 454 reads map to a total of 2,545 gene models that were not covered previously, and identified 73 additional alternative NAGNAGs. Even though the 454 data cover only 75% (3,745/5,031) of the NAGNAGs evidenced by Sanger ESTs, they enabled detection of 371 alternative NAGNAGs - 9.9% of the covered NAGNAGs, as compared to 7.5% using Sanger ESTs. Of these 371, 117 were previously identified by Sanger ESTs. There are 42 NAGNAGs which have support for only one acceptor in the Sanger data, and for only the other acceptor in 454 data. Combining the results from Sanger and 454 data, *P. patens *has 664 alternative NAGNAGs. Again these results show that NAGNAG AS is as widespread in the moss *P. patens *as it is in the seed plants *A. thaliana *and *O. sativa*. An overview of the NAGNAG sites covered by the different transcript evidence pools is presented in Table 3.

**Table 3:** Summarized NAGNAG coverage

**Sanger-based NAGNAG sites**				
	**covered NAGNAG sites**	**EI form**	**E form**	**I form**
**Sanger ESTs**	5,031	295	2,695	2,041
**454 reads**	3,745	371	1,974	1,400
**combined evidences**	5,031	591	2,549	1,891
**Additional 454-based NAGNAG sites**				
	**covered gene models**	**EI form**		
**454 reads**	2,545	73		
**Total alternative NAGNAG sites**				
		664		

### Experimental confirmation of the NAGNAG AS

Experiments were performed on 19 candidate NAGNAGs, 14 as controls (seven with AS according to transcript data, and seven without AS) to see whether the splicing outcomes according to Sanger and 454 reads could be confirmed by a PCR based approach, and five on the basis of an orthologous alternative NAGNAG intron in *A. thaliana *(see above). Of the seven candidates with support for AS from Sanger or 454 datasets, three were predicted to be alternative spliced with p(EI) values > 0.9 (Additional file 2). Using Sanger sequencing of cDNA based PCR products, all three candidates were indeed verified as being alternatively spliced in *P. patens *protonema and gametophore tissue, respectively. Eight candidate genes were used as potential negative controls, as their p(EI) predictions were 0.365 and lower. All candidates showed support for the single predicted isoform by means of available transcript evidence and consequently only this single isoform could be detected during experimental validation (Additional file 2). Having support for both variants from either the Sanger or the 454 datasets, but a p(EI) < 0.9, four more candidates were chosen to be validated. NAGNAG AS could be confirmed for the gene product Phypa_161321 by Sanger sequencing of cDNA PCR products, although it has a low p(EI) of 0.181 (Additional file 2). The experimental validation is supported by the Sanger dataset, where 13 "E" variants as well as 27 "I" variants could be identified. This is the only case where prediction from the Naïve Bayes Classifier does not agree with the experimental results. In case of Phypa_74146 and Phypa_199161, only one of the two isoforms could be detected, reflecting the low p(EI) values.

**Figure 4:**
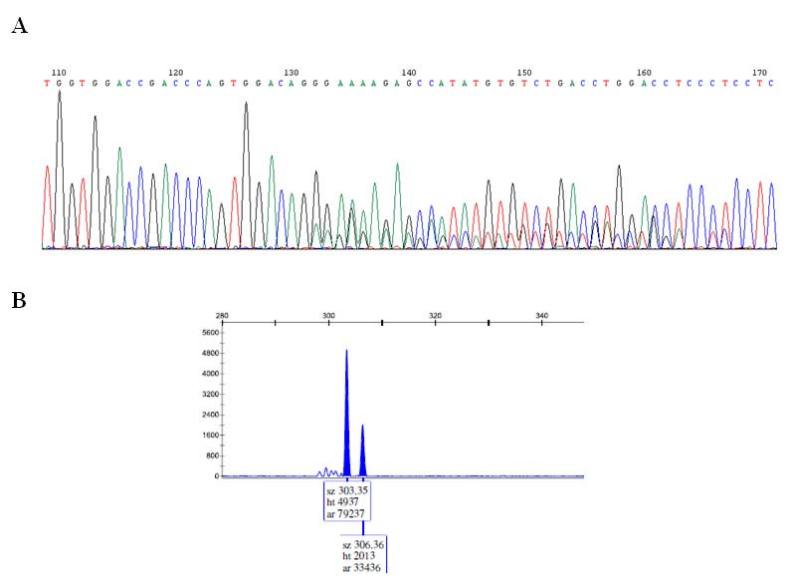
**Example of the validation procedures employed**. A: Electropherogram of the direct sequencing of a cDNA PCR product. Starting with the NAGNAG AS site at position 132 the two alternatively spliced sequence signatures are overlaid. B: FAM fluorescence intensity peaks of the two splice variants (length difference three nucleotides). The lower peak constitutes 40% of the area (ar) of the bigger one, i.e. an approximate ratio of 3:1.

The sensitivity of Sanger sequencing allows detection of AS for ratios of the two isoforms of about 3:1 or lower, meaning that cases in which the minor isoform abundance is < 25%, AS may go undetected even if present. Therefore, validation using fluorescence labeled forward primers and fragment length detection on a capillary sequencer was used to detect the minor isoform abundance for two examples. In case of Phypa_161321 (Additional file 2) the received data determined by Sanger sequencing of PCR products could be confirmed by the more sensitive detection using the fluorescence labeled forward primers. The two isoforms with three nucleotides difference in length were detected using capillary separation and had a relative abundance of approximately 3:1 (exonic "E" versus intronic "I" variant) (Figure 4). In case of Phypa_228333, only one of the two isoforms could be detected by Sanger sequencing as well as in the more sensitive validation using fluorescence labeled primers (Additional file 2). Thus, a low p(EI) prediction for this candidate seems to be correct as is the case for Phypa_74146 and Phypa_199161, for which only one of the two isoforms could be identified as predicted. Detection of both isoforms either in Sanger datasets (Phypa_199161) or in the 454 datasets (Phypa_74146 and Phypa_228333) could be explained by the higher sensitivity of sequencing as compared to the PCR-based approach or by the fact that adult gametophores were used to generate the 454 data, while the validation was carried out in the two principal tissues of the juvenile stage. Thus it cannot not be excluded that these candidates are indeed alternatively spliced.

### GAG acceptors

Twelve of the 19 candidate genes possess a GAG in the NAGNAG motif (Additional file 2). Using the above described methods, all of them are shown to be not alternatively spliced. Therefore, GAG seems not to be used as an alternative acceptor for AS in *P. patens *in most cases, which is in line with the sequence logos (Figure 2B). Exceptions could be Phypa_199161 and Phypa_228333, which possess both isoforms regarding Sanger and 454 datasets. These two candidates may indeed use GAG as acceptors for AS, but this remains to be proven. Rare usage of GAGs as acceptors in *P. patens *is in agreement with previous work which shows that functional acceptors are only very rarely GAGs - the order of preference for the nucleotide preceding the AG in functional acceptors is C > T > A > G, which has been shown both by experimental work [31] as well as by *in silico *analyses of NAGNAG splicing [5,12]. When we consider the EST and 454 evidence in *P. patens*, only 4.6% (149/3225) of GAG-containing NAGNAGs are alternative - filtering by transcript support to use only well-supported cases (as described for the preparation of training data in the "Methods" section) further reduces this to 2.6% (14/536). Taken together, this strongly suggests that GAGs function only very rarely as functional acceptors in *P. patens *(if at all).

### Using 454 data for independent validation of predictions

The classifier was trained based on previously existing Sanger evidence, the additional 454 evidence was used for independent validation. Combining the 454 and Sanger datasets resulted in 296 additional NAGNAG AS events being detected - of these, 66 had strong support for AS in terms of satisfying the criteria used to define the training dataset (≥ 2 reads for each variant, ≥ 10% of the reads for the minor variant). 62% (41/66) of these were predicted to be alternative by the Naïve Bayes classifier. If we require ≥ 4 reads per variant while keeping the threshold of minor variant abundance at ≥ 10%, the correct predictions rise to 75% (9/12). When considering AS according to 454 reads alone, 64% (80/125) of the well-supported cases of AS are predicted correctly, which increases to 79% (30/38) if we require ≥ 4 reads per variant while keeping the threshold of minor variant abundance at ≥ 10%. On the other hand, if we look at cases which are constitutive with a support of ≥ 30 transcripts, according to the combined transcript dataset, only 1/93 E cases and 0/65 I cases are predicted to be alternative. The Naïve Bayes classifier predicts 371 further cases of AS (155 of 2,549 currently labeled E, and 216 of 1,891 currently labeled I) in *P. patens *- the high specificity shown by nearly no predicted AS in strongly supported constitutive NAGNAGs combined with the sensitivity of 62% in detecting newly discovered strongly supported cases of AS shows that there are potentially several hundred as yet undiscovered cases of NAGNAG AS in *P. patens*.

### Prediction of NAGNAG AS in *P. patens *by a classifier trained on *H. sapiens *data

We had earlier shown that a classifier trained on only *H. sapiens *NAGNAG data could predict NAGNAG splicing outcomes with near-identical accuracy on other vertebrate genomes (mouse, rat, dog, chicken), and with a slight drop in the case of *D. melanogaster *and *Caenorhabditis elegans *[10]. Therefore, we also tried to predict NAGNAG AS in *P. patens *using a Naive Bayes classifier trained on *H. sapiens *data and achieved an AUC of 0.90, 0.99 and 0.97 for the EI, E and I forms, respectively. This was achieved using five features (the Ns in the NAGNAG, the two positions immediately upstream and the position immediately downstream) and is similar to that achieved on *D. melanogaster *earlier [10], reinforcing the notion that NAGNAG splicing in plants is similar to that in animals.

## Conclusions

Here we describe the first computational prediction of alternative splicing (AS) in a non-seed plant and find that NAGNAG AS in *P. patens*, a moss, can be predicted with high accuracy. Since the extent of NAGNAGs in *P. patens *had not yet been reported, this work involved both characterization as well prediction of NAGNAG splicing in *P. patens*. Using ESTs, we found that NAGNAG AS is as widespread in the bryophyte *P. patens *as it is in the seed plants *A. thaliana *and *O. sativa*. Thus, NAGNAG AS is likely to be a common feature of AS in all land plants, just as it is in animals. Although we detected homologs with NAGNAG events among the two land plants *P. patens *and *A. thaliana*, NAGNAG splicing seems not to be conserved at the intron level.

Using carefully constructed training and test datasets, an *in silico *performance of AUC = 0.96, 0.99 and 0.98 was achieved for the EI, E and I forms, respectively. The most informative features (according to information gain [32]) were the nucleotides in the NAGNAG and its immediate vicinity, and even a relatively simple classifier like the Naïve Bayes classifier could match the more sophisticated Bayesian network and Support vector machine. The performance achieved by a Naïve Bayes classifier trained on *H. sapiens *data (AUC = 0.90, 0.99 and 0.97 for the EI, E and I forms, respectively) was similar to that achieved on *D. melanogaster *earlier [10]. This indicates that, as in animals, the mechanism behind NAGNAG AS in plants is simple in nature and mostly dependent on the splice site neighborhood. Independent validation of the predictions of the classifier (trained on Sanger EST data alone) using 454 data showed that 64% (80/125) of the well-supported cases of NAGNAG AS could be predicted correctly.

In total, seven candidates were chosen for independent experimental confirmation of the Sanger and 454 evidence of NAGNAG splicing. The experimental confirmation depends on detection of isoforms using sequence electropherograms and is less sensitive than size polymorphism detection using fluorescence-labeled primers. The latter method was used on two of the seven examples and confirmed the results of the previous method. While there is transcript support for alternative use of GAG acceptors this could not be proven in our experimental validation. In addition, a further 12 experiments were performed - six as negative controls, all of which agreed with the predictions, and five to check for possible conserved NAGNAG AS with *A. thaliana*, which could not be detected.

When additional 454 transcript evidence was used to supplement the Sanger EST data, a total of 664 alternative NAGNAGs were found in *P. patens*. Since the average coverage per constitutive NAGNAG was still only approximately ten ESTs, this number shall likely continue to rise with deeper coverage of the transcriptome. Nevertheless, the results provide the first evidence that NAGNAG AS is widespread in *P. patens*. Our findings are in agreement with a recent study which showed that NAGNAG AS shares common properties in *A. thaliana *and *O. sativa *and animals [24]. This indicates that the mechanism behind NAGNAG AS in land plants is similar to that in animals. The pervasiveness of NAGNAG AS suggests that it may be a general feature of splicing in animals and plants, and possibly in all eukaryotes.

## Methods

### Identification of alternative splicing at NAGNAG acceptors using ESTs

**Figure 5:**
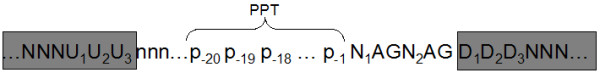
**Nomenclature of features used in this study**. Nomenclature of sequence features used to analyze NAGNAG splicing. The region used to derive all 31 features is shown, along with the names given to the positional features.

346,871 *P. patens *Sanger EST reads (available at http://www.cosmoss.org) from various developmental stages and tissue types (predominantly protonema and juvenile gametophores) were aligned using GenomeThreader [33]. EST alignments (max. intron length 20,000) with less than 95% identity and 90% EST length coverage were excluded from further analyses to obtain only reliable alternative acceptors. In addition, EST alignments matching a single exon as well as alignments ending at an exon boundary supporting either the E or I site were discarded. The sequence regions used for feature extraction (Figure 5) and EST evidence counts were created using the BioPerl [34] module Bio::DB::SeqFeature::Store.

### Sequence logos

Sequence logos were created using the WebLogo software http://weblogo.berkeley.edu/logo.cgi[35] with the sequence regions shown in Figure 5.

### Feature design and extraction; classifiers

Feature extraction was done based on annotated data using a Perl script (Additional file 3; see Additional file 4 for example input data. The script produces output which, together with Additional file 5, can be used with standard classifiers). The region used for analysis can be seen in Figure 5. Since the composition of the splice site neighborhood influences splicing in general, the base pairs at positions -20 to +3 with respect to the NAGNAG were each used as a single feature, as were the two Ns in the NAGNAG motif. The last three positions of the upstream exon were also included, since they can influence both the process of splicing, as well as reflect influence of codon usage near the exon boundary. Thus, we had a total of 28 features which each represented a nucleotide, and thus had four possible values (A, C, G, T). A weak polypyrimidine tract (PPT) can contribute to AS, and the number of pyrimidines in the 3' region of the intron is a measure of PPT strength. Therefore, we designed a feature called "Y-content", which refers to the number of pyrimidines in the 20 bp upstream of the NAGNAG. Splice site strength, being one of the most important determinants of splicing outcome, was also included as a feature - the strength of the two possible splice sites for each NAGNAG exon, as computed using SpliceMachine [36], contributed two more features. In total, 31 features were used. We used the WEKA package and Bayesian Networks, Naive Bayes classifiers, and Support vector machines [32]. For feature selection within WEKA, we used the method "CfsSubsetEval". In addition, we also used manual inclusion and exclusion of features.

### Information gain

Information gain is defined as the reduction in the entropy of the class variable, given the feature [32]. The formula for information gain is:

where H(Class) is the entropy of the class variable, and H(Class|Feature) is the conditional entropy of the class variable, given the feature. Information gain is a well established measure for feature selection in Machine Learning. We used the WEKA package for computing information gain, in order to rank the features according to how informative they were. We also used it for prediction based on SVMs, as implemented in the SMO option, and for prediction using Naïve Bayes classifiers.

### Functional annotation and GO enrichment analysis

For every (potential) NAGNAG splicing region an overlapping *P. patens *gene model was assigned using the start and stop coordinates on the genomic scaffolds. The corresponding predicted protein sequences were subjected to BLAST2GO [37] GO term annotation which was extended by various subcellular target prediction and homology-based methods (see http://www.cosmoss.org/annotation/references?cosmoss_ref=1 for details). The resulting GO annotation was mapped to GO slim terms using the Blast2GO internal mapping function using the "goslim_plant.obo" ontology subset. GO enrichment analysis was performed against the complete *P. patens *with the BLAST2GO internal Fisher's exact test/GOSSIP [38] using the two-tailed test, with false discovery rate (FDR) correction and a q-value cut-off < 0.05. The *A. thaliana *alternative NAGNAG splicing gene set was constructed using the alternative NAGNAG acceptor cases identified within the *A. thaliana *genome from [26] and [24]. The resulting alternative NAGNAG acceptor set contains 290 *A. thaliana *proteins. These proteins were subjected to a GO enrichment analysis as described above for *P. patens*. The *A. thaliana *GOA was downloaded from ftp://ftp.arabidopsis.org/home/tair/Ontologies/Gene_Ontology/ATH_GO_GOSLIM.txt (17.11.2009) and mapped to GO slim (goslim_plant.obo) with BLAST2GO.

### Candidate selection for evolutionary conserved NAGNAG acceptors

*P. patens *cosmoss v1.2 and *A. thaliana *TAIR 8 proteins were subjected to a BLAST based single linkage clustering using BLASTCLUST [39]. The parameters were set to 70% length coverage and 70% alignment identity to obtain only highly conserved homologs. In total 1,088 clusters with at least one *P. patens*, respectively *A. thaliana*, protein were found. Five candidates out of seven *P. patens *genes, each sharing a cluster with *A. thaliana *alternative NAGNAG acceptor containing genes [24,26], were selected for experimental validation. In addition, these *P. paten*s candidate genes contain a potential NAGNAG acceptor in the same intron as the corresponding *A. thaliana *homolog.

### Experimental confirmation of splice variants

*P. patens *total RNA was isolated from protonema and gametophore tissue using the RNeasy Plant Mini Kit (Qiagen, Hilden, Germany). cDNA synthesis was carried out with 250 ng total RNA using Superscript III Reverse Transcriptase (Invitrogen, Karlsruhe, Germany) according to the manufacturers' instructions. For validation of different splice variants, PCR was performed from protonema and gametophore RNA, respectively, using native *Pfu*-Polymerase (Fermentas, St. Leon-Rot, Germany). PCR primers were obtained from Sigma (München, Germany). PCR reactions were carried out using 12 ng cDNA as template. Products were extracted using the QIAquick PCR purification Kit (Qiagen, Hilden, Germany) and directly sequenced (GATC, Konstanz, Germany). Sequences and chromatograms were analysed with ChromasPro Version 1.34. Alternatively, PCR products amplified with carboxyfluorescein (FAM) labeled forward primers were analysed by capillary electrophoresis, where AS was detected as a size difference of three nucleotides in length. PCR products were diluted as appropriate and subjected to capillary electrophoresis for separation and detection. For this purpose, 10 μL HiDi formamide (Applied Biosystems) and 0.5 μL HD400 GS internal size standard were added to each well, and the plate was mounted on a 3100 Genetic Analyzer with Foundation Data Collection software v. 2.0 and Gene Mapper ID software v. 3.2 (Applied Biosystems, Darmstadt, Germany).

### Tissue culture and generation of additional transcript evidence

*Physcomitrella patens *strain Gransden 2004 [23] was cultivated on solidified (1% w/v agar) mineral medium [250 mg L-1 KH_2_PO_4_, 250 mg L^-1 ^MgSO_4 _× 7-H_2_O, 250 mg L^-1 ^KCl, 1000 mg L^-1 ^Ca(NO_3_)_2 _× 4H_2_O, 12.5 mg L^-1 ^FeSO_4 _× 7H_2_O, pH 5.8 with KOH] on 9 cm petri dishes enclosed by laboratory film in a Percival cultivation chamber (CLF, Germany) at 22°C with a 16 h light, 8 h dark regime under 70 μmol*s^-1^*m^-2 ^white light (long day conditions). Gametophore colonies were grown from single gametophores transferred to the dishes from precultured colonies. Induction of gametangia was performed by placing the dishes under inductive conditions [40], i.e. 20 μmol *s^-1^*m^-2 ^white light and 15°C with a 8 h light, 16 h dark regime until development of gametangia. After harvesting and freezing, the material was ground under liquid nitrogen and total RNA isolated using the Ambion mirVana miRNA isolation kit (Applied Biosystems, Darmstadt, Germany). RNA isolation and subsequent sequencing pool creation steps were carried out by Vertis Biotechnologie (Freising, Germany). Poly(A)+ RNA was prepared by oligo(dT) chromatography and cDNA was synthesized using a N6 randomized primer. Afterwards, 454 adapters A (CCATCTCATCCCTGCGTGTCTCCGACTCAG) and B (CTGAGACTGCCAAGGCACACAGGGGATAGG) were ligated to the 5' and 3' ends of the cDNA. The resulting N0 cDNA was amplified using PCR (16 cycles) with a proof reading enzyme. Normalization was carried out by one cycle of denaturation and reassociation of the cDNA, resulting in N1-cDNA. Reassociated ds-cDNA was separated from the remaining ss-cDNA (normalized cDNA) by passing the mixture over a hydroxylapatite column. After hydroxylapatite chromatography, the ss-cDNA was amplified with 9 PCR cycles. Finally, the cDNA in the size range of 500-700 bp was eluted from a preparative agarose gel and subjected to GS FLX Titanium sequencing (GATC, Konstanz, Germany), resulting in 631,313 raw reads. After low quality and adapter clipping using LUCY [41] and SeqClean http://compbio.dfci.harvard.edu/tgi/software/, and polyA-tail removal with trimmest [42], 589,283 reads with a mean length of 343 nucleotides remained. The 454 reads (Additional file 1) were mapped against the genome as described above for the *P. patens *Sanger ESTs and are available at http://www.cosmoss.org for download in a genome browser track "454 reads sexual gametophores (normalized library)" http://www.cosmoss.org/cgi/gbrowse/physcome/

## Authors' contributions

RS analyzed the transcript evidences for NAGNAGs, designed and extracted features, used WEKA to perform the classification and analyses, computed the genome-wide prediction, and drafted the manuscript. ADZ obtained the transcript evidences for all genomic NAGNAGs in *P. patens*, helped in feature extraction, performed the GO-analysis, and contributed to writing the manuscript. KB performed the experimental validations and contributed to writing the manuscript. DL participated in the transcript evidence analyses, and helped with the GO-analysis. RR and MP supervised part of the work. RB and SAR contributed to writing the manuscript, conceived of and supervised the study. All authors have read and approved the final manuscript.

## Supplementary Material

Additional file 1 **All NAGNAGs in *Physcomitrella patens***. Information on all 5,031 NAGNAGs in *Physcomitrella patens*, including gene name, EST name, genomic location, transcript support, and the predictions of the Naïve Bayes classifier. Click here for file

Additional file 2 Summarized experimental results. Click here for file

Additional file 3 **Perl script**. The Perl script used for feature extraction. Click here for file

Additional file 4 **Transcript support data**. The transcript support data derived from the http://www.cosmoss.org database. Click here for file

Additional file 5 **Names file**. Names file for combination with the script output. Click here for file
