# Supplementary material for: Identification and characterization of NAGNAG alternative splicing in the moss Physcomitrella patens
Source: BMC Plant Biol. 2010 Apr 28;10:76. doi: 10.1186/1471-2229-10-76 (PMC3095350; doi:10.1186/1471-2229-10-76)
Supplement: Additional file 2 — Summarized experimental results. [file 1471-2229-10-76-S2.DOC]

Table 1 Summarized experimental results

|  |  |  |  |  |  |  |  |  |  |  |  |  |  |  |  |  |  |  | **sequence length polymorphism** | | |
| --- | --- | --- | --- | --- | --- | --- | --- | --- | --- | --- | --- | --- | --- | --- | --- | --- | --- | --- | --- | --- | --- |
| **gene id** | **P(EI) NBC** | **P(E) NBC** | **P(I) NBC** | **P(EI) BN** | **P(E) BN** | **P(I) BN** | **Sanger_E** | **Sanger_I** | **454_E** | **454_I** | **transcript support for EI** | **GAG** | **A.t. homolog with AS** | **fwd sequence 5'--->3'** | **rev sequence 5'--->3'** | **start** | **end** | **product size** | **gametophore** | | **protonema** |
| **High P(EI) + transcript support** | | | | | | | | | | | | | | | | | | | | | |
| Phypa_193990 | 0.940 | 0.000 | 0.060 | 0.969 | 0.013 | 0.018 | 4 | 20 | 4 | 8 | x |  |  | TGTTGGGGAAGTTTTGAAGG | CATCCTCGTCTTTGTGTTCG | 113 | 469 | 356 | yes | | yes |
| Phypa_80579 | 0.912 | 0.000 | 0.088 | 0.896 | 0.101 | 0.003 | 3 | 2 | 6 | 2 | x |  |  | ACCCTCGGTCTTCTACTCGAC | TGTAGCTCCAGGCTCAGTCTC | 860 | 1164 | 304 | yes | | yes |
| Phypa_106363 | 0.901 | 0.006 | 0.093 | 0.500 | 0.025 | 0.475 | 9 | 7 | 8 | 8 | x |  |  | GCGTCTTGTGGCACCTTTAG | GGGTAGGCGCATGTCTTTAC | 4 | 335 | 331 | yes | | yes |
| **Low P(EI) + transcript support** | | | | | | | | | | | | | | | | | | | | | |
| Phypa_161321 | 0.181 | 0.819 | 0.000 | 0.196 | 0.804 | 0.000 | 13 | 27 | 1 | 0 | x |  |  | CGGTGGCTATGTGGTCATC | CTGACGGCATCACACAAGAC | 728 | 1035 | 307 | yes | | yes |
| *Phypa_161321_fwd_FAM* |  |  |  |  |  |  |  |  |  |  | x |  |  | CGGTGGCTATGTGGTCATC |  |  |  |  | yes | | yes |
| Phypa_74146 | 0.177 | 0.000 | 0.823 | 0.063 | 0.001 | 0.936 | 0 | 4 | 1 | 10 | x |  | x | GGATCTCTTCTCTGCGATGC | ACCAGATGAAGAACAAGATTGC | 27 | 332 | 305 | no | | no |
| Phypa_199161 | 0.015 | 0.000 | 0.985 | 0.122 | 0.000 | 0.877 | 2 | 16 | 0 | 0 | x | x |  | GTGGTACAATTGCCGATTCC | AGAGTCAGCTCATCGCCAAC | 447 | 747 | 300 | no | | no |
| Phypa_228333 | 0.000 | 1.000 | 0.000 | 0.000 | 1.000 | 0.000 | 97 | 0 | 10 | 2 | x | x |  | ACAATCGGTGCTGAATCTCC | GAGTAAGGATGGCGTTCTCC | 223 | 589 | 366 | no | | no |
| *Phypa_228333_fwd_FAM* |  |  |  |  |  |  |  |  |  |  | x | x |  | ACAATCGGTGCTGAATCTCC |  |  |  |  | no | | no |
| **Low P(EI) + no transcript support** | | | | | | | | | | | | | | | | | | | | | |
| Phypa_100961 | 0.085 | 0.915 | 0.000 | 0.019 | 0.981 | 0.001 | 14 | 0 | 13 | 0 |  |  |  | CTGGCTACTTCGGAGGTGAC | ACAGAGCTGAGGTGGTCTGG | 278 | 659 | 381 | no | no | |
| Phypa_117470 | 0.000 | 1.000 | 0.000 | 0.000 | 1.000 | 0.000 | 12 | 0 | 7 | 0 |  | x |  | TCAAGCTCTTCGAGGTTTCC | ATGTCGAAACGCTGCATAAC | 1415 | 1718 | 303 | no | no | |
| Phypa_181992 | 0.000 | 1.000 | 0.000 | 0.000 | 1.000 | 0.000 | 39 | 0 | 4 | 0 |  | x |  | ATACCCAGATCGGTGTTTCG | GCTGGTACCCTTCTGCAATG | 303 | 628 | 325 | no | no | |
| Phypa_181992 | 0.001 | 0.000 | 0.999 | 0.000 | 0.000 | 1.000 | 0 | 11 | 0 | 3 |  | x |  | ACATTTGGACAGGGTTACCG | TGACTTCAACACGTCCTTCG | 1342 | 1651 | 309 | no | no | |
| Phypa_171213 | 0.000 | 1.000 | 0.000 | 0.000 | 1.000 | 0.000 | 43 | 0 | 33 | 0 |  | x |  | CTGCTGCCACAGACTTCCTC | TTCTTCACCTTCTTCCTGTCG | 34 | 378 | 344 | no | no | |
| Phypa_145753 | 0.000 | 1.000 | 0.000 | 0.008 | 0.991 | 0.000 | 64 | 0 | 5 | 0 |  | x |  | TGAAGCATTGCAAAGAGTGC | CAACACTCCTTGGCTGGAAC | 92 | 429 | 337 | no | no | |
| Phypa_114834 | 0.190 | 0.809 | 0.001 | 0.032 | 0.967 | 0.001 | 1 | 0 | 3 | 0 |  | x |  | TGATCCAAGGCTACTGATTGC | CTATGGGCGATCATGTGAAG | 636 | 938 | 302 | no | no | |
| Phypa_121999 | 0.312 | 0.687 | 0.001 | 0.572 | 0.425 | 0.003 | 2 | 0 | 5 | 0 |  | x |  | CGACTGAGAACAAATTCGAAAG | GTTGGCTCAGAGGATGGTTC | 11 | 322 | 311 | no | no | |
| **Homologous intron AS in *A. thaliana*** | | | | | | | | | | | | | | | | | | | | | |
| Phypa_180723 | 0.001 | 0.999 | 0.000 | 0.001 | 0.999 | 0.000 | 1 | 0 | 0 | 0 |  | x | x | GTTTGCCTCGGAGATGAAAG | AGGCCAACACAGAAGGAGTG | 480 | 821 | 341 | no | no | |
| Phypa_180457 | 0.001 | 0.999 | 0.000 | 0.001 | 0.999 | 0.000 | 1 | 0 | 1 | 0 |  | x | x | CTTGCCTCTGTGGGAGTGTC | GTGCAGAATCAGCAACATCC | 108 | 453 | 345 | no | no | |
| Phypa_216093 | 0.005 | 0.995 | 0.000 | 0.029 | 0.968 | 0.002 | 2 | 0 | 9 | 0 |  | x | x | GGGAATTGGTTGATGTGACG | CCTACCACTTCCATCGGTTC | 10 | 333 | 323 | no | no | |
| Phypa_191544 | 0.365 | 0.635 | 0.000 | 0.734 | 0.266 | 0.000 | 5 | 0 | 14 | 0 |  |  | x | AGCCAGTCGCTTAGATCTGG | ATTCCCTCCAAATCCTCCAC | 257 | 596 | 339 | no | no | |
